# Modeling Task Uncertainty for Safe Meta-Imitation Learning

**DOI:** 10.3389/frobt.2020.606361

**Published:** 2020-11-27

**Authors:** Tatsuya Matsushima, Naruya Kondo, Yusuke Iwasawa, Kaoru Nasuno, Yutaka Matsuo

**Affiliations:** ^1^School of Engineering, The University of Tokyo, Tokyo, Japan; ^2^DeepX Inc., Tokyo, Japan

**Keywords:** meta-learning, imitation learning, robot learning, task uncertainty, safety

## Abstract

To endow robots with the flexibility to perform a wide range of tasks in diverse and complex environments, learning their controller from experience data is a promising approach. In particular, some recent meta-learning methods are shown to solve novel tasks by leveraging their experience of performing other tasks during training. Although studies around meta-learning of robot control have worked on improving the performance, the safety issue has not been fully explored, which is also an important consideration in the deployment. In this paper, we firstly relate uncertainty on task inference with the safety in meta-learning of visual imitation, and then propose a novel framework for estimating the task uncertainty through probabilistic inference in the task-embedding space, called PETNet. We validate PETNet with a manipulation task with a simulated robot arm in terms of the task performance and uncertainty evaluation on task inference. Following the standard benchmark procedure in meta-imitation learning, we show PETNet can achieve the same or higher level of performance (success rate of novel tasks at meta-test time) as previous methods. In addition, by testing PETNet with semantically inappropriate or synthesized out-of-distribution demonstrations, PETNet shows the ability to capture the uncertainty about the tasks inherent in the given demonstrations, which allows the robot to identify situations where the controller might not perform properly. These results illustrate our proposal takes a significant step forward to the safe deployment of robot learning systems into diverse tasks and environments.

## 1. Introduction

The development of generalist robots remains a key challenge in robotics. These robots are expected to perform a wide range of tasks in diverse environments, for instance, automation of household chores or operations in retail stores. As hand-engineering specific skills for every task and environment is clearly infeasible, many studies focus on data-driven approaches, which learn desired skills from experiences. For example, reinforcement learning is a popular choice, where robots learn their control policies from the interaction with the environment (Kober et al., 2013; Sutton and Barto, 2018). In addition, imitation learning is a more sample-efficient strategy in robot learning, which leverages demonstrations collected by experts or via teleoperation (Pomerleau, 1989; Hussein et al., 2017; Zhang et al., 2018). From the perspective of learning task-general robot controllers, meta-learning is among the most prominent new methods in transfer learning, in which the algorithm learns to adapt to new environments or tasks (Finn et al., 2017a). For these reasons, meta-imitation learning is considered to be a powerful robot learning method and several studies have focused on it to date. Especially, MIL (Finn et al., 2017b) and TecNets (James et al., 2018) learn policies using behavioral cloning, which enables offline learning. Both models learn to adapt to new tasks by inferring the task using demonstrations at hand and performing with a controller conditioned on the inferred task. The way each model adapts is different—MIL uses gradient-based adaptation and TecNets uses conditional policies based on an explicit context variable.

Although the performance when adapting to various tasks is aimed at in previous studies, in the deployment of the robot system, the safety must also be considered. From the viewpoint of the safety of learned robot controllers, some studies work on ensuring safety in reinforcement learning (Garcıa and Fernández, 2015; Lötjens et al., 2019) and imitation learning (Zhang and Cho, 2016; Thakur et al., 2019). The settings in which robots have been deployed thus far are usually single-task and relatively simple, therefore we can imagine the behaviors that robots may perform in advance, and take measures that will ensure the safety of the robots and their environments. The safety of generalist robots, however, can be different from those conventional ones, in that there is no guarantee that the task given at test time can be achievable with the learned controller. Furthermore, if a robot is deployed in an environment with large variations in the tasks it must perform, it becomes impossible to anticipate and model behavior when a task inference is mistaken, which may lead to unsafe conditions in the real world. To give an example of meta-imitation learning, without any safety conditions, the robot may perform wrong with confidence even when the demonstration given to a robot is improper or ambiguous to specify the task requested at test time.

Keeping this concern in mind, we found that studies in meta-imitation learning to date implicitly suppose that the demonstrations given to robots during the test phase will be clear enough that the robot can be certain about the task at hand, while the demonstrations can potentially be noisy during real deployment. One possible solution to this issue is to measure the uncertainty on task inference from the demonstration, or the uncertainty about which task to perform, which we refer to as *task uncertainty*. When the task uncertainty is high, the task inference could be wrong. The robot could then take measures to ensure the safety of its action; for example, one measure could be leveraging the fallback policy by requesting additional demonstrations of the task to be performed. It should be noted that while some studies on imitation learning consider the uncertainty of the controller, these discussions are restricted to single-task settings. We instead focus on meta-imitation learning, and we are not aware of discussions about uncertainty in task inference for meta-imitation learning in the literature so far.

Considering the discussion above, this paper focuses on capturing task uncertainty from demonstrations in meta-imitation learning problems. We propose a new algorithm, Probabilistic Embedding over Task-space Network (PETNet), which embeds task information for adapting the controller as a distribution. This algorithm is intended to measure task uncertainty while deploying a meta-imitation learning algorithm. PETNet is designed around the notion that as a demonstration can sometimes be ambiguous, task embedding should be modeled in a probabilistic manner. This probabilistic inference on the task variable allows us to quantify how certain the model is about the task from the demonstrations at hand. As for the implementation, the model simultaneously learns the task embedding network that infers explicit task variables along with the control policy conditioned on those task variables. The task embedding network outputs a distribution on the task embedding space from few-shot demonstrations and we use the variance of the distribution as the measure of task uncertainty in these demonstrations.

We tested PETNet with the simulated manipulation environment presented in Finn et al. (2017b) and confirm that it can achieve state-of-the-art-level performance in the standard benchmark. We then show that the evaluated uncertainty in PETNet is useful for detecting demonstrations that are inappropriate for task identification. Tests were run with the demonstrations in the original dataset and with synthesized out-of-distribution demonstrations. In addition, the performance degrades if we use demonstrations with high task uncertainty, and increasing the number of demonstrations for adaptation can help reduce the task uncertainty. These results show that PETNet can contribute to the safety of meta-imitation learning for controllers by preventing robots from behaving unexpectedly when the demonstrations have too much uncertainty for robots to identify the task.

To summarize, key contributions of this paper are (1) proposing a framework for leveraging uncertainty of task indicated with demonstrations for realizing the safety of robot under various tasks in meta-imitation learning and (2) with the simple implementation with probabilistic inference on task embedding space, this approach can capture the task uncertainty, leading to identify demonstrations with which the model would not perform properly.

## 2. Related Work

Most studies aiming at generalist robots have focused on improving the sample efficiency of the learning algorithm. As in other domains in machine learning, such as image (Zamir et al., 2018) and natural-language processing (Devlin et al., 2019), transfer learning is one of the promising directions for efficient training of robot control to perform various tasks. For example, much of the literature in robot learning utilizes the sim-to-real approach. These algorithms train a general policy on simulated and diverse environments and then this policy is deployed to the real environment of interest (Tobin et al., 2017; Peng et al., 2018; James et al., 2019). By leveraging simulated data, which is easier to obtain compared to real-world datasets, the sim-to-real approach sometimes successfully learns a policy that works well even in the real environment; however, it requires a great deal of effort in the design of simulator environments.

Other than learning one general policy as in sim-to-real approaches, several recent works focus on how to quickly adapt a policy to a new task of interest, which is in general referred to as meta-learning. Meta-learning is explained as a method to learn meta-knowledge to decide model bias depending on the target task or domain using experience (Vilalta and Drissi, 2002). This approach is general across machine learning and is not limited to robot control. In models that use neural networks, the meta-learner adapts to test settings by either changing network parameters (Finn et al., 2017a) or inferring latent variables (Garnelo et al., 2018). A substantial number of studies have addressed meta-learning of robot control. For example, recurrent network (Duan et al., 2016) and gradient descent (Finn et al., 2017a,b) have been used for adaptation of the policy network, while James et al. (2018), Queißer and Steil (2018), Rakelly et al. (2019), and Seyed Ghasemipour et al. (2019) utilize conditional policies with latent variables. Since the policy needs to both identify the test environment or task and adapt to it, we can expect better performance with a specific meta-learned policy. In this paper, our model is based on the idea of meta-learning and we propose a method that can estimate task uncertainty using probabilistic inference to latent task variables.

As for the algorithm used for policy learning, popular choices include reinforcement learning or imitation learning. Reinforcement learning is based on reward signals (Sutton and Barto, 2018). Finn et al. (2017a), Humplik et al. (2019), and Rakelly et al. (2019) focus on the intersections of meta-learning and reinforcement learning. Instead of learning from scratch, imitation learning, also referred to as learning from demonstrations (Schaal, 1997; Argall et al., 2009), utilizes demonstrations presented to the robots in the form of teleportation (Van Den Berg et al., 2010), kinesthetic teaching (Kober and Peters, 2009), or as a demonstration video performed by a human (Sermanet et al., 2018; Yu et al., 2018). While this paper is based on imitation learning because it improves the sample efficiency of interactions with the environment, the core ideas discussed below can be extended to other learning methods in which measuring task uncertainty is useful for safe deployment of robot learning. Inverse reinforcement learning and behavioral cloning are the main algorithms in imitation learning. In the former method, the algorithm learns to recover the reward function behind demonstrations by assuming they are optimal under the reward function (Ng and Russell, 2000). Behavioral cloning, in contrast, is a supervised method by which the algorithm learns mappings from observation to actions directly manner (Pomerleau, 1989). In the context of meta-imitation learning, behavioral cloning is often used (Finn et al., 2017b; James et al., 2018), while Seyed Ghasemipour et al. (2019) and Xu et al. (2019) adopt inverse reinforcement learning. Our method employs behavioral cloning for the sake of its algorithmic simplicity. Note again that none of the studies above on meta-learning of robot control emphasize the importance of task uncertainty for ensuring safety.

Some papers mention the significance of evaluating predictive uncertainty to ensure the safety of the controller in the context of imitation learning. Previous papers have pointed out that the main problems in imitation learning lies in the inherent ambiguity of demonstrations (Goo and Niekum, 2019) or the discrepancy between training and test conditions that can lead robots to perform unexpected actions (Pomerleau, 1989; Osa et al., 2018). In practice, one possible solution is measuring the predictive uncertainty, and if the robots are uncertain about their prediction, they can stop performing actions and request that experts provide additional demonstrations (Thakur et al., 2019). These studies above, however, only consider single-task settings and the task uncertainty has never been taken into consideration yet, while our work is rather focusing on the task uncertainty of meta-learning and the safety of the controller.

## 3. Measuring Task Uncertainty in Meta-Imitation Learning

**Figure 1:**
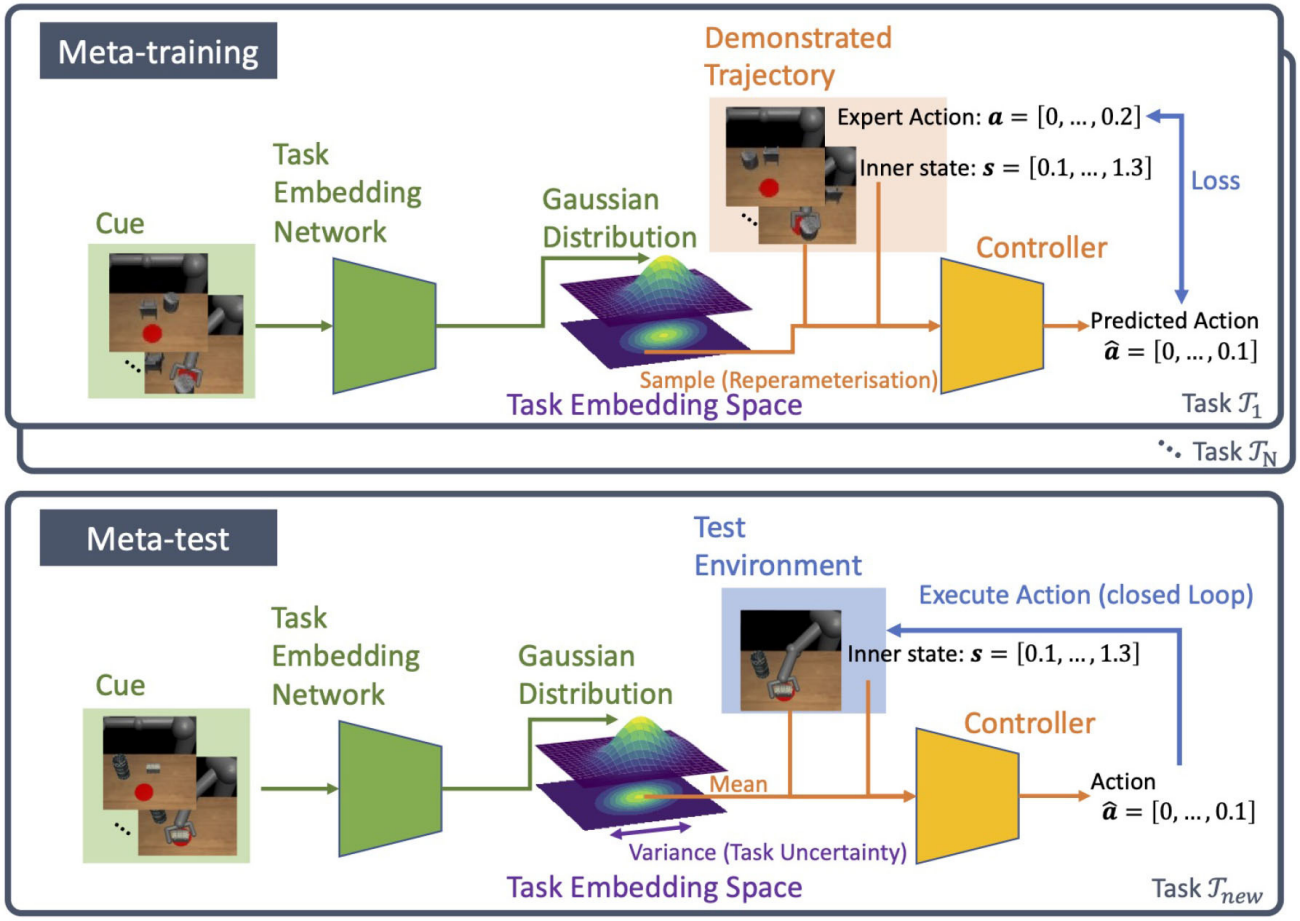
Overview of PETNet. The model consists of two neural networks, task embedding network and controller. During meta-training, the latent task variable is sampled from the distribution over task embedding. In the meta-test, the variance is the measurement of task uncertainty.

Our model PETNet consists of two parts, the task embedding network and the controller. The task embedding network builds a distribution over the task embedding space from visual demonstrations of the task. In this paper, the distribution is Gaussian with mean and variance as the output of the network. The controller is modeled with another neural network and takes the robot's observation and the task variable as inputs. The controller functions in a closed-loop as shown in Figure 1.

### 3.1. Problem Statement of Meta-Imitation Learning

The goal of meta-imitation learning is to obtain a policy that can be adapted for use in a new task given one or a few demonstrations (Finn et al., 2017b). The policy π maps observations ***o*** to predicted actions $\hat{a}$. Each imitated task is defined as

(1) $$\begin{array}{r} T_{i}=\left\{\tau =\left\{{\text{o}}_{1},{\text{a}}_{1},\ldots ,{\text{o}}_{T},{\text{a}}_{T}\right\}\sim \pi_{i}^{⋆},L\left({\text{a}}_{1:T},{\hat{\text{a}}}_{1:T}\right),T\right\}, \end{array}$$

where τ is the demonstration generated by expert policy $\pi_{i}^{⋆}$ and L is the loss function. We assume that each task $T_{i}$ is sampled from the task distribution p(T).

During meta-training, the robot is presented two types of demonstrations from expert $\pi_{i}^{⋆}$ about the task $T_{i}$. One is for adapting the model to the task that we refer as the *cue* $\tau_{i}^{\left(c\right)}$, and one is for measuring the loss of the policy. We refer to the latter as the *demonstrated trajectory* $\tau_{i}^{\left(t\right)}$. During meta-testing, for a new task $T_{new}\sim p\left(T\right)$, the robot performs in the actual environment provided only with one or a few cues $\tau_{new}^{\left(c\right)}$ from expert $\pi_{new}^{⋆}$.

In this paper, we use only sequences of visual observation for the cues $\tau_{i}^{\left(c\right)}$, so actions are not included, since in some problem settings, robots cannot access expert actions for cue demonstrations, for example, a human expert performs the cue as in Yu et al. (2018). For the demonstrated trajectory $\tau_{i}^{\left(t\right)}$, we use a sequence of observations and the actions of the robot in each timestep.

### 3.2. Probabilistic Task Embedding

The main focus of this paper is to propose a method that can be deployed ensuring safety in the setting of meta-imitation learning. For this purpose, PETNet adopts a simple approach that measures the uncertainty of task inference from cues $\tau_{new}^{\left(c\right)}$. If the task uncertainty is modeled, we can expect the safety to be enhanced, because, for example, the robot can avoid performing unexpectedly during meta-testing caused by an uncertain task inference.

In concrete, the task embedding network $f\left(\tau_{i}^{\left(c\right)};\theta \right)$ infers a latent task variable as an explicit random variable in a stochastic manner. This network provides the distribution over embedding $p\left(z_{i}|\tau_{i}^{\left(c\right)}\right)$ conditioned by the cue $\tau_{i}^{\left(c\right)}$ by outputting the mean and variance of the distribution, that is,

(2) $$\begin{array}{r} p\left(z_{i}|\tau_{i}^{\left(c\right)}\right)=N\left(\mu_{i}^{\left(c\right)},\sigma_{i}^{2\left(c\right)}\right)\;\text{where}\;\mu_{i}^{\left(c\right)},\sigma_{i}^{2\left(c\right)}=f\left(\tau_{i}^{\left(c\right)};\theta \right). \end{array}$$

The controller π(***o***, ***z***_*i*_; **ϕ**) takes the task variable ***z***_*i*_ and the visual observation ***o*** as inputs.

The network architecture we used in the experiments is almost the same as the previous method TecNets (James et al., 2018), in concrete, both the task embedding network and the controller have four convolutional and three fully-connected layers. The difference in PETNet is that the controller has two outputs, namely the mean and variance of the Gaussian task embedding distribution, while in TecNets, the output is the task variable alone, which is passed directly into the controller. See section 4.1.1 for details of the implementation.

The task variable ***z***_*i*_ is sampled from the task embedding distribution during meta-training as $z_{i}\sim p\left(z_{i}|\tau_{i}^{\left(c\right)}\right)$. As we hypothesize that the distribution is Gaussian, we can use reparameterization tricks in the sampling procedure to train both the task embedding networks and the controller simultaneously. To train the model, we use the mean-squared error between the action from the demonstrated trajectory and the predicted action from the policy π with the parameter **ϕ**, so the loss for task $T_{i}$ is

(3) $$\begin{array}{r} L_{i}=\frac{1}{T}\underset{\left(o,a\right)\in \tau_{i}^{\left(t\right)}}{\sum }∥\pi \left(o,z_{i};\phi \right)-a{∥}_{2}^{2} \end{array}$$

where *T* denotes the number of timesteps. The full training procedure is given in Algorithm 1.

During meta-testing, when the robot faces with the new task $T_{new}$ determined by given cue demonstrations $\tau_{new}^{\left(c\right)}$, the task variable ***z***_*new*_ is computed as the mean of $p\left(z_{new}|\tau_{new}^{\left(c\right)}\right)$. Then the controller outputs its action â_*t*_ = π(***o***_*t*_, ***z***_*new*_; **ϕ**) for each timestep *t*. The variance from the task-embedding network is the measurement of the uncertainty about inference from the cue to the task. Algorithm 2 describes the procedure used in meta-testing.

The cue is not restricted, in principle, to videos of demonstrations performed by the same robot. The cue could be any demonstration, like a video of a human performing an action, as long as the task information is preserved. We will discuss the generalizability of the approach further in the section 5.

**Algorithm 1:** Meta-training of PETNet.

**Require**: Task distribution p(T)
1: **while** not done **do**
2: Sample batch of tasks ${\left\{T_{i}\right\}}_{i=1}^{N}\sim p\left(T\right)$
3: **for all** $T_{i}$ **do**
4: Sample cue $\tau_{i}^{\left(c\right)}\sim T_{i}$ and demonstrated trajectory $\tau_{i}^{\left(t\right)}\sim T_{i}$
5: Encode demos $\mu_{i}^{\left(c\right)},\sigma_{i}^{2\left(c\right)}=f\left(\tau_{i}^{\left(c\right)};\theta \right)$
6: Sample task embedding $z_{i}\sim N\left(\mu_{i}^{\left(c\right)},\sigma_{i}^{2\left(c\right)}\right)$
7: Compute loss for task $T_{i}$ as $L_{i}=\frac{1}{T}\underset{\left(o,a\right)\in \tau_{i}^{\left(t\right)}}{\sum }∥\pi \left(o,z_{i};\phi \right)-a{∥}_{2}^{2}$
8: **end** **for**
9: Compute average loss $L=\frac{1}{N}\overset{N}{\underset{i=1}{\sum }}L_{i}$
10: Update parameters **θ**, **ϕ** with gradient descent
11: **end** **while**

**Algorithm 2:** Meta-test of PETNet.

**Require**: Cue $\tau_{new}^{\left(c\right)}$ of meta-test (hold-out) task $T_{new}\sim p\left(T\right)$
Encode demos $\mu_{new}^{\left(c\right)},\sigma_{new}^{2\left(c\right)}=f\left(\tau_{new}^{\left(c\right)};\theta \right)$
Use $\sigma_{new}^{2\left(c\right)}$ as measure of task uncertainty
Use $\mu_{new}^{\left(c\right)}$ as task embedding $z_{new}=\mu_{new}^{\left(c\right)}$
**for** *t* = 1… *T* **do**
***o***_*t*_ = *Env*.*GetObservation*()
***â***_*t*_ = π(***o***_*t*_, ***z***_*new*_; **ϕ**)
*Env*.*Act*(***â***_*t*_)
**end** **for**

## 4. Experiment

In this section, we explain the experimental tests and our analysis of the characteristics of the PETNet algorithm. These tests address the following questions:

1. Can PETNet attain performance comparable to that of previous methods in meta-testing?
2. Does PETNet can capture the uncertainty of the task embedding inferred from the cue?
3. Is the uncertainty estimated by PETNet useful for improving the safety of the controller?

### 4.1. Experimental Setting

We evaluate PETNet with a simulated pushing task of MIL sim-push dataset using the MuJoCo physics simulator (Todorov et al., 2012)^1^. The task is to push the target object indicated by the demonstration to the designated goal area using a 7-DoF robot arm.

Each task varies in terms of the objects in the environment and the target object to push. Within the same task, the pair of objects and the relationship of the target and the distractor is fixed, but their arrangement in the environment changes. 769 tasks are used for meta-training including those for validation. 74 tasks are held out for meta-testing, each of which has different objects than the meta-training tasks. Although 24 demonstrations are included for each task in the original MIL sim-push dataset, we used 12 demonstrations for each task following the experimental setting of previous studies, with six tasks for the cue and six for the demonstrated trajectory. The setup of disjoint datasets, which consists of the cues and demonstrated trajectories, comes from the motivation of meta-imitation learning, which aims to adapt to a new task given some information about the task at hand from the cue, while the demonstrated trajectories are used to measure the loss and train the policy. Therefore, the cues and demonstrated trajectories do not have to be in the same domain [for example, Yu et al. (2018) uses human video of a task as the cue and trajectories of target robot for the corresponding task as the demonstrated trajectory]. In our experiment, the cues and demonstrated trajectories are performed in the same setting (the same robot, camera, etc.), which is the simplest case we can consider.

A successful trial is defined as an episode in which the robot places the target object inside the goal area for at least 10 timesteps in a 100-timestep episode. The average success rate of six trials is reported for all meta-testing tasks.

#### 4.1.1. Implementation Details

The visual demonstration is a series of 125 × 125 RGB images with 100 timesteps. The sequence is represented by concatenating the first and last frame channel-wise, which we found is sufficient for MIL sim-push dataset (we may use an RNN if we are more interested in representing the dynamics). The network architecture is almost the same as TecNets for fair comparison. Both task embedding network and the controller have four convolutional layers (CNN) followed by three fully-connected layers. Each convolutional layer has 5 × 5 filters of 16 channels with 4 strides and ReLU activation function (Nair and Hinton, 2010) is applied. The proprioceptive data (the joint angles, joint velocities, and end-effector pose of the robot arm) is concatenated to the output of the last layer of the CNN. The fully-connected layer has 200 units, except for the last layer. The number of output of the task embedding network is 20, 10 for the mean, and 10 for the variance of Gaussian distribution, which results in the same number of parameters as TecNets (TecNets uses task embedding vector with the length of 20). The output of the controller is torques applied to each joint of the 7DoF robot arm. We used Adam optimizer (Kingma and Ba, 2015) with the learning rate of 5.0 × 10^−4^.

### 4.2. Performance of Simulated Pushing Task

We compared the performance of PETNet with that of two previous methods, namely MIL (Finn et al., 2017b) and TecNets (James et al., 2018). Table 1 is the success rate in the meta-test of the simulated pushing task. PETNet attained a success rate of 72.52%, which outperforms MIL with visual demonstrations (66.44%) and is comparable to the performance of TecNets ($\lambda_{ctr}^{U}$ = 0) (70.72%). It should be noted that although the main result of TecNets (main) (77.25%) is obtained by using actions from both the cues and the demonstrated trajectories, PETNet uses actions from the demonstrated trajectories only. This selection is more general as a setting in meta-imitation learning and it is fairer to compare PETNet with the ablation version of TecNets, namely TecNets ($\lambda_{ctr}^{U}$ = 0). TecNets (λ_*emb*_ = 0) is the variant without the cosine embedding loss during the learning and its performance is worse than the other variants (58.56%), which shows the loss term is necessary to group embeddings of the same task in TecNets. However, while PETNet does not have such explicit embedding loss term, the performance is almost the same as TecNets, which implies that the probabilistic inference-based method of PETNet can contribute appropriate task representation in the embedding space. As shown in the experiment above, the proposed method for estimating task uncertainty has no negative impact on the final performance of meta-imitation.

**Table 1:** Performance at meta-test of sim-push task.

**Method**	**Success Rate (%)**
MIL (vision)	66.44
TecNets (main)	77.25
TecNets ($\lambda_{ctr}^{U}$ = 0)	70.72
TecNets (λ_*emb*_ = 0)	58.56
PETNet (ours)	72.52

*The numbers of MIL and TecNets are taken from the original papers. TecNets (main) uses both internal states and visual observations as the cue demonstrations, whereas other methods use visual observation only. TecNets (λ_emb_ = 0) is a variant of TecNets without the cosine embedding loss*.

### 4.3. Analysis of Task Embedding and Task Uncertainty

Next, we analyzed the characteristics of the learned representation of the task embedding from the demonstrations. The focuses of this analysis is to find whether the task embedding effectively captures the uncertainty in the inference from cue to task, and to find whether this measure of uncertainty is useful for ensuring robot safety.

#### 4.3.1. Visualization of Task Embedding

**Figure 2:**
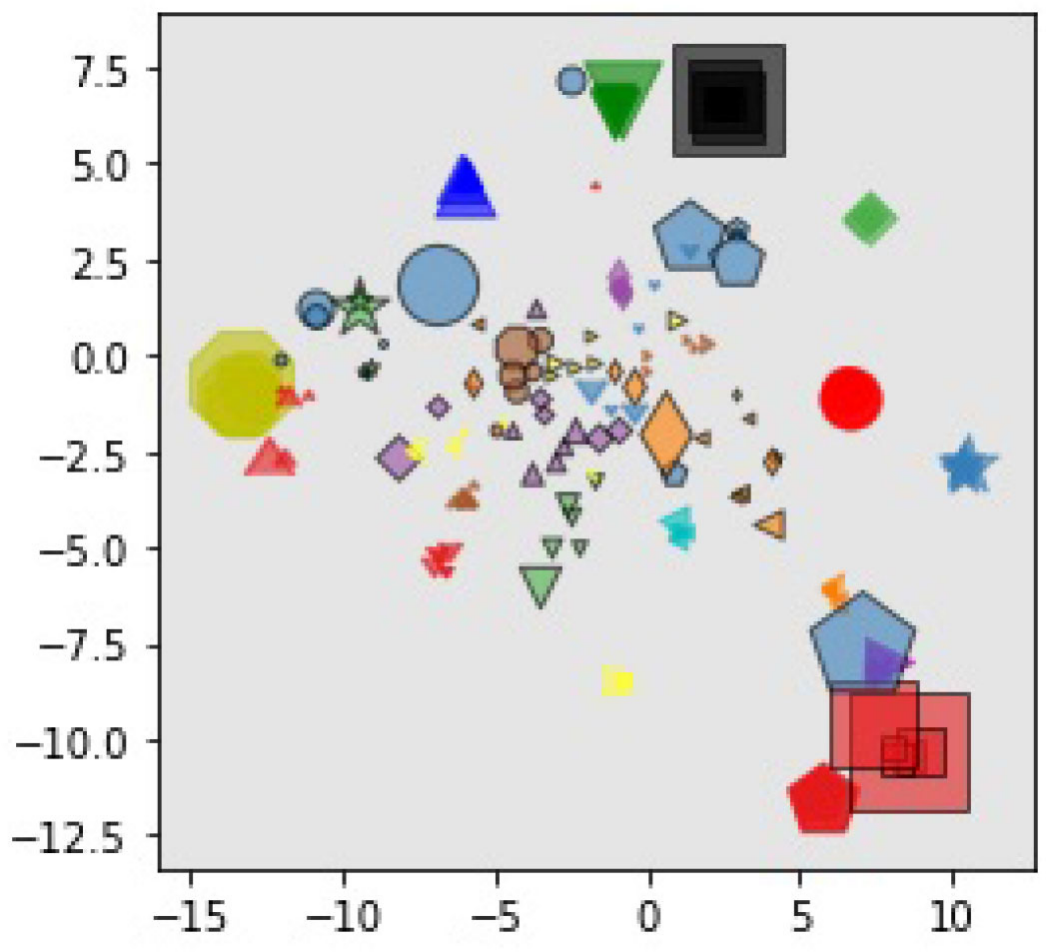
Visualization of task embedding for randomly selected demonstrations, which is reduced to two-dimensional space with t-SNE. Points with the same marker denote demonstrations from the same task and markers with black round means demonstrations in meta-test tasks. The size of each marker is proportional to the variance of the task embedding.

Firstly, we visualized the task embedding by reducing its dimensions using t-SNE (van der Maaten and Hinton, 2008). As illustrated in Figure 2, we chose 15 tasks randomly from the meta-training and meta-testing tasks and we plotted the mean of each task embedding distribution. The same mark (shape and color) corresponds to the same task but the demonstration is different. The marker size is proportional to the variance of the task embedding. Meta-testing tasks are highlighted with a black border around the mark. Although we did not introduce any explicit loss function for the embedding, we found that the embeddings of the same task are grouped together for both meta-training tasks and meta-testing tasks, suggesting that the task representation learned by this model was generalized even for meta-test tasks.

#### 4.3.2. Evaluating Task Uncertainty

We then conducted three experiments to evaluate whether PETNet can correctly estimate task uncertainty to detect inappropriate demonstrations for imitation learning.

**Figure 3:**
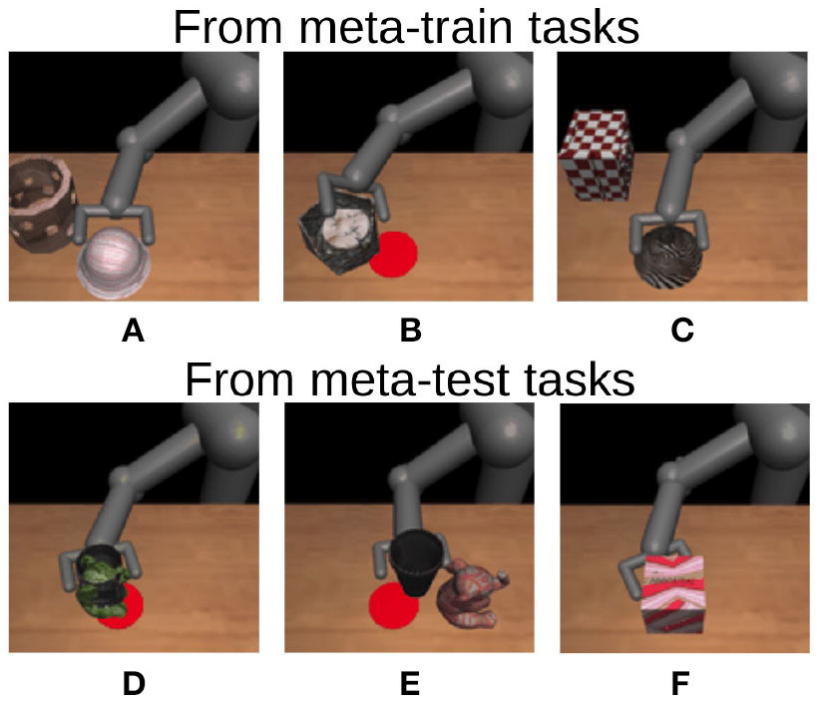
Demonstrations with highest variances in task embedding space of PETNet. Demonstrations in the first row are from meta-training tasks, while those in the second row are from meta-test tasks. These demonstrations can be inappropriate for task identification for the controller, for example, in **(A–E)**, the arm pushes both target object and distractor, especially in **(B)** and **(D)**, they are overlapping, and in **(F)**, the target object is completely occluded by the other.

Firstly, we applied the learned task embedding network to the original MIL pushing dataset. Figure 3 shows some of the top 13 demonstrations with the highest variance of the task embedding distribution. The demonstrations in the first row are from meta-training tasks and those in the second row are from meta-testing tasks. While the MIL pushing dataset Finn et al. (2017b) seems well-controlled in its data generation, PETNet identifies some demonstrations that are inappropriate for task identification using the task embedding network. For example, the robot pushes both objects in (B) and (D), and the target object is completely occluded by the other in (F). This result shows that PETNet can find semantically inappropriate demonstrations without explicit supervision.

**Figure 4:**
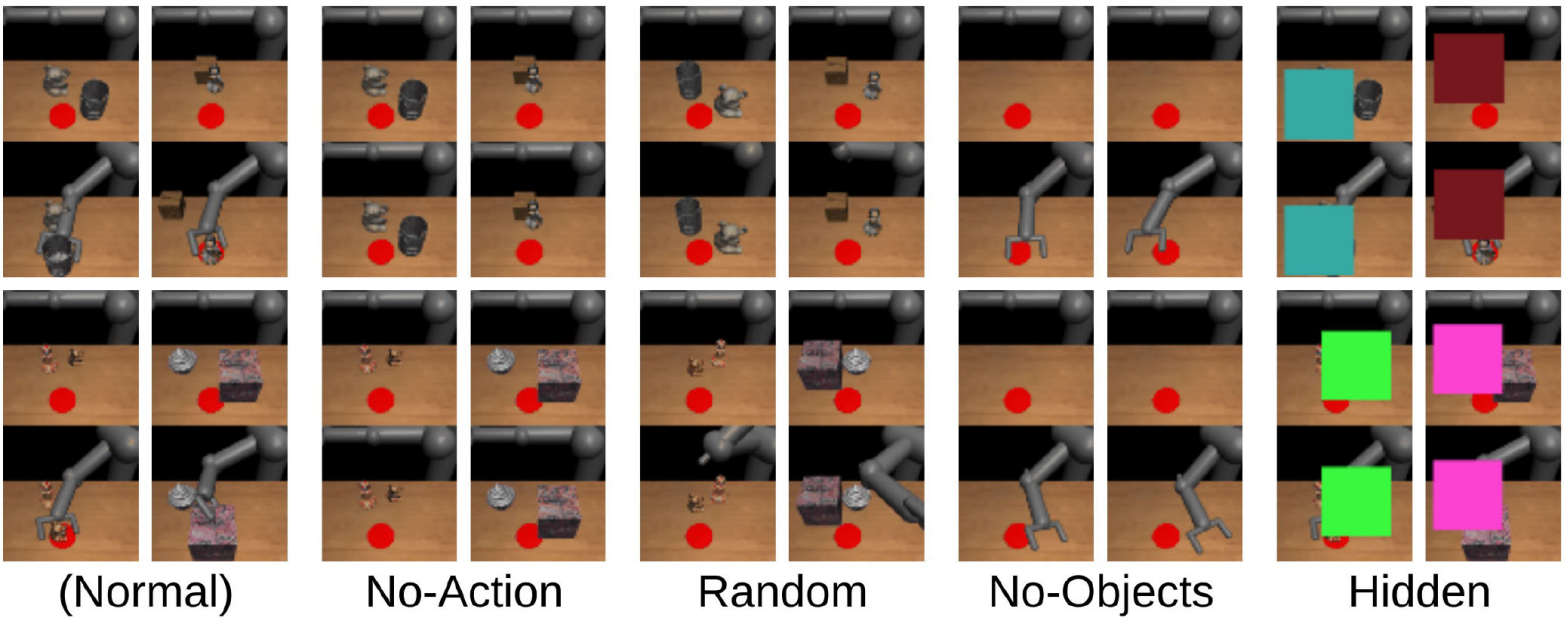
Examples of synthesized out-of-distribution demonstrations (the normal demonstrations are from the original datasets).

**Figure 5:**
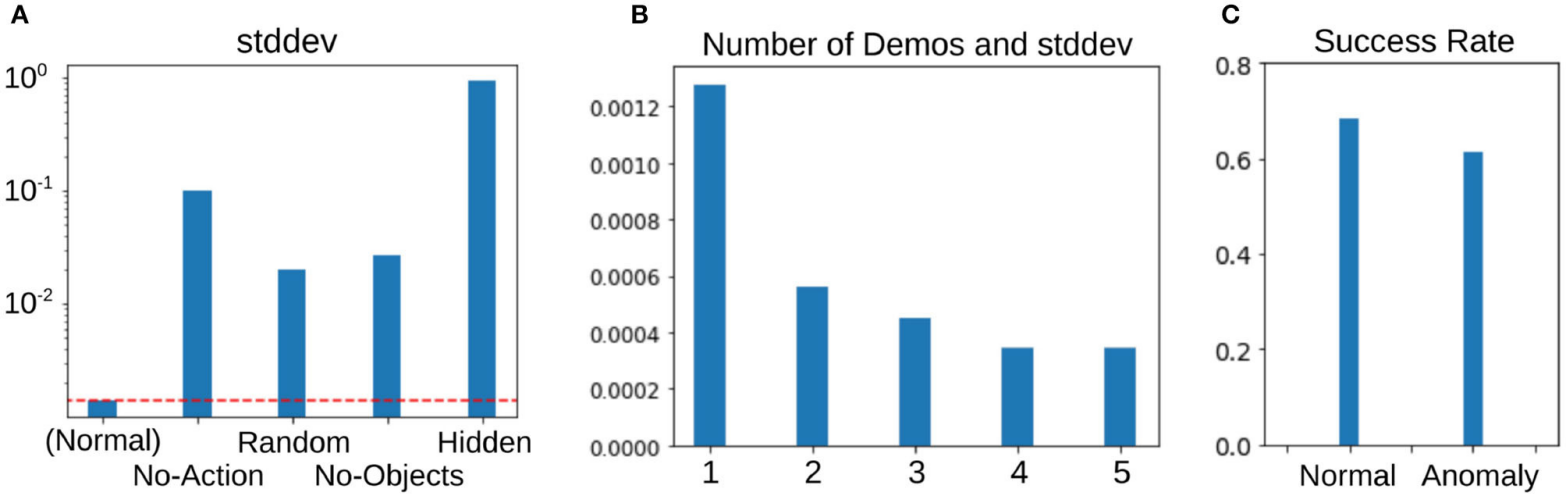
Experimental results about task uncertainty. **(A)** Average standard deviation of the task-embedding distribution using the original and synthesized out-of-distribution demonstrations as in Figure 4. **(B)** Average standard deviation with different number of demonstrations. **(C)** Comparison of performance using the demonstrations with normal and anomalous standard deviations during meta-testing.

Secondly, we synthesized some out-of-distribution demonstrations and checked whether PETNet can detect those examples that are not included in the original dataset and have no information about the task. Figure 4 represents the examples of the demonstrations. Starting on the left, the demonstrations in the original dataset (Normal), the robot arm does not move (No-Action), the robot arm moves randomly (Random), no objects are in the environment (No-Objects), and the demonstration is hidden by squares randomly placed in the observation (Hidden). Figure 5A shows the average standard deviation of the task embedding distribution for each category of demonstration. The standard deviations for synthesized out-of-distribution demonstrations are obviously larger than those using demonstrations in the original dataset, implying that PETNet correctly detects the out-of-distribution demonstrations. This is an essential property for removing demonstrations with large task uncertainty during deployment.

Thirdly, we evaluated the standard deviation changing the number of demonstrations during meta-testing. This assessment assumes that the robot can access several demonstrations for each task, rather than only one demonstration in other settings. The task embedding distribution from multiple demonstrations is represented as a product of each distribution from a single demonstration and we can compute the product analytically, as in Hinton (2002). The result is shown in Figure 5B and confirms that increasing the number of demonstrations reduces task uncertainty.

#### 4.3.3. Task Uncertainty and Performance

If the task identification fails with the given cue in meta-imitation learning, the controller will not be able to attain the goal of the task and may perform unexpectedly. Therefore, it is necessary to know how certain the task identification is before the execution for preventing such undesired failure. We show in the previous section that the variance of the distribution over the task embedding space is useful for finding inappropriate cues for identifying the task (e.g., occlusion or object overlapping). Here, we can hypothesize that we can prevent such failures if the inappropriate cues are detected by monitoring the variance.

Therefore, we examined the relationship between the task uncertainty of the demonstrations and performance during meta-testing. We employ anomaly detection on the estimated variances to detect the anomalous demonstrations that may cause failure, using a one-class support vector machine (OCSVM) with a linear kernel (Schölkopf et al., 2001). This is the most basic choice for unsupervised anomaly detection. The classifier is trained with the variances of the task embedding distribution from training tasks only, assuming that they contain 1% anomalous demonstrations. During meta-testing, we detect anomalous demonstrations from the variances of the demonstration using this linear OCSVM model. Figure 5C clearly shows that the task performance with the cues detected as anomalies is worse than the normal one by about 10%, which suggests that the task execution is more likely to fail if we use such anomaly cues and that the anomaly detection can actually work for preventing failures (we confirmed that the variances of those anomalies have always contained some large element and that the model detects those high variances).

## 5. Discussion and Conclusion

In this paper, we first pointed out the significance of evaluating task uncertainty in meta-learning for robot control to ensure safe deployment. Motivated by this insight, we proposed PETNet, a novel method for measuring the task uncertainty in meta-imitation learning using probabilistic task embedding. We showed that PETNet achieves better performance than previous methods in the benchmark of the simulated pushing task, and tracks the uncertainty of the task while performing it. This feature could be important to ensure the safe deployment of meta-imitation learning algorithms applied to broader ranges of tasks.

The evaluations presented in this paper are done with the MIL dataset using a physics simulator, which is a standard setting for meta-imitation learning. However, deploying robot learning methods into real robots is not as easy as simulators and there can be several challenges. For example, if we think about transferring policies in simulators to real environments, sim-to-real is itself a challenge in robot learning, because we have to tackle the domain gap between simulator and real environment. On the other hand, when learning only from real environments, another concern arises about how to make the model “task-efficient” as well as the sample-efficient, because it is costly to prepare various tasks and their demonstrations. In addition, the “task” in the experiments is defined as the combination of the target object and the distractor, and the aspect of “pushing an object” is shared in all tasks. Scaling meta-imitation learning to different “skill-level” tasks (i.e., pushing, pick-and-place, peg insertion, etc.) is the ongoing work in the robot learning community (Yu et al., 2019, 2020; James et al., 2020). Concurrently, since Ajay et al. (2020) reports that their model learns these skill embedding based on probabilistic inference, we suppose that our framework can scale to more diverse tasks, which we would like to evaluate as future work.

As the extensions of our work, the approach of measuring task uncertainty on the task-embedding space can be generalizable to other learning algorithms like reinforcement learning or inverse reinforcement learning. Our results open the door for using demonstrations in other modalities like language instructions, human demonstrators (Yu et al., 2018) or multiple demonstrators via crowdsourcing services (Mandlekar et al., 2018) which can inherently have higher uncertainty in task identification. Leveraging the evaluated task uncertainty for meta-training (for example, weighing imitation learning loss according to task uncertainty) could also be an important extension of our work and could also take the form of curriculum learning or active learning, which optimizes both data-collection and control policies.

## Data Availability Statement

Publicly available datasets were analyzed in this study. This data can be found at: https://rail.eecs.berkeley.edu/datasets/mil_data.zip.

## Author Contributions

TM designed the study, developed the method, and wrote the initial draft of the manuscript. NK contributed to the implementation of the code, analysis of data, and assisted in the preparation of the manuscript. YI contributed to the experimental design and has critically reviewed the manuscript. KN and YM contributed to the design of the study and preparation of the manuscript. All authors contributed to the article and approved the submitted version.

## Conflict of Interest

KN was employed by the company DeepX Inc. The remaining authors declare that the research was conducted in the absence of any commercial or financial relationships that could be construed as a potential conflict of interest.

## Footnotes

^1^The dataset is available in the original codebase of MIL at https://github.com/tianheyu927/mil.
